# Large Vessel Vasospasm Is Not Associated with Cerebral Cortical Hypoperfusion in a Murine Model of Subarachnoid Hemorrhage

**DOI:** 10.1007/s12975-018-0647-6

**Published:** 2018-07-12

**Authors:** Axel Neulen, Simon Meyer, Andreas Kramer, Tobias Pantel, Michael Kosterhon, Svenja Kunzelmann, Hermann Goetz, Serge C. Thal

**Affiliations:** 1 Department of Neurosurgery, University Medical Center of the Johannes Gutenberg-University, Langenbeckstrasse 1, 55131 Mainz, Germany; 2 Department of Anesthesiology, University Medical Center of the Johannes Gutenberg-University, Langenbeckstrasse 1, 55131 Mainz, Germany; 3 Platform for Biomaterial Research, University Medical Center of the Johannes Gutenberg-University, Langenbeckstrasse 1, 55131 Mainz, Germany

**Keywords:** Subarachnoid hemorrhage, Cerebral hypoperfusion, Cerebral vasospasm, Microcomputed tomography, Laser SPECKLE contrast imaging

## Abstract

Clinical studies on subarachnoid hemorrhage (SAH) have shown discrepancies between large vessel vasospasm, cerebral perfusion, and clinical outcome. We set out to analyze the contribution of large vessel vasospasm to impaired cerebral perfusion and neurological impairment in a murine model of SAH. SAH was induced in C57BL/6 mice by endovascular filament perforation. Vasospasm was analyzed with microcomputed tomography, cortical perfusion by laser SPECKLE contrast imaging, and functional impairment with a quantitative neuroscore. SAH animals developed large vessel vasospasm, as shown by significantly lower vessel volumes of a 2.5-mm segment of the left middle cerebral artery (MCA) (SAH 5.6 ± 0.6 nL, sham 8.3 ± 0.5 nL, *p* < 0.01). Induction of SAH significantly reduced cerebral perfusion of the corresponding left MCA territory compared to values before SAH, which only recovered partly (SAH vs. sham, 15 min 35.7 ± 3.1 vs. 101.4 ± 10.2%, *p* < 0.01; 3 h, 85.0 ± 8.6 vs. 121.9 ± 13.4, *p* < 0.05; 24 h, 75.3 ± 4.6 vs. 110.6 ± 11.4%, *p* < 0.01; 72 h, 81.8 ± 4.8 vs. 108.5 ± 14.5%, n.s.). MCA vessel volume did not correlate significantly with MCA perfusion after 72 h (*r* = 0.34, *p* = 0.25). Perfusion correlated moderately with neuroscore (24 h: *r* = − 0.58, *p* < 0.05; 72 h: *r* = − 0.44, *p* = 0.14). There was no significant correlation between vessel volume and neuroscore after 72 h (*r* = − 0.21, *p* = 0.50). In the murine SAH model, cerebral hypoperfusion occurs independently of large vessel vasospasm. Neurological outcome is associated with cortical hypoperfusion rather than large vessel vasospasm.

## Introduction

Aneurysmatic subarachnoid hemorrhage (SAH) is a form of hemorrhagic stroke and presents a frequent clinical picture in neurointensive care [1–3]. While the hemorrhage itself and associated global cerebral hypoxia induce fatal brain injury in some patients, clinical outcome in patients surviving the initial hemorrhagic event is thought to be largely determined by cerebral hypoperfusion and large vessel vasospasm (CV) [1–6], which occur in the days and weeks following the bleeding event due to pathophysiological events induced by the subarachnoid hematoma [4]. However, more recent clinical studies have reported discrepancies between CV, cerebral hypoperfusion, and neurological outcome [7–11], attenuating the association of CV with an unfavorable outcome.

Similar to the disease in humans, mice develop CV and impaired cerebral perfusion after experimental induction of SAH [12–22]. Although these changes occur with faster temporal dynamics in mice than humans—peaking 3 days after SAH in mice [16, 23, 24] compared to 7 to 10 days in humans [3]—murine models have become an important tool for basic research on the pathophysiology of SAH. The majority of experimental studies to date have focused on CV [12–20]. By contrast, little data on cerebral perfusion in mice after SAH have been published [20]. Furthermore, the relation between CV, cerebral hypoperfusion, and neurological deficits in murine SAH models remains unclear despite the fact that, from the clinical perspective mentioned above [5–10], cerebral hypoperfusion may be the most important parameter concerning unfavorable outcome. Thus, better knowledge of the link between vasospasm, hypoperfusion, and neurological deficits in the murine model could benefit future experimental studies. In the present study, we set out to (i) characterize cerebral perfusion in a murine endovascular filament perforation model of SAH, (ii) assess the contribution of CV to changes in cerebral perfusion, and (iii) investigate whether neurological disability is linked to CV and changes in cerebral perfusion.

## Methods

### Ethics, Animals, and Housing Conditions

The animal experiments were approved by the responsible animal care committee (Landesuntersuchungsamt Rheinland-Pfalz, G12-1-093) and carried out in accordance with the German Animal Welfare Act (TierSchG). All applicable international, national, and institutional guidelines for the care and use of animals were followed.

We used male C57BL6 mice (Charles River, Cologne, Germany; age, 11–14 weeks). No other inclusion or exclusion criteria were defined. Mice were kept under controlled environmental conditions (12-h dark/light cycle, 23 ± 1 °C, 55 ± 5% relative humidity) with free access to food (Altromin, Germany) and water. Body weight was recorded daily as a general marker of well-being. A neuroscore (0 to 29 points, with 0 indicating no neurological deficit and 29 indicating severe neurological disability) was determined 1 day prior to and 24 and 72 h after SAH or sham surgery, as previously described [25, 26]. Neuroscores were determined by an investigator blinded to the treatment.

In a separate set of experiments (data not shown), we observed that (i) mean arterial pressure measured daily before and 3 days after SAH did not differ significantly between SAH and sham groups and that (ii) intracranial pressure (ICP), after its peak during SAH induction, returned to nearly baseline levels only 3 h post-insult, indicating that cerebral perfusion pressure was similar between SAH and sham animals at 3, 24, and 72 h after SAH induction. These findings were consistent with previous reports [27].

### Anesthesia and Murine Model of SAH

Measurements of cerebral perfusion, SAH induction, and transcardiac perfusion were performed in anesthetized animals. Anesthesia was induced with 4% (*v*/*v*) isoflurane for 1 min and maintained with 2% (*v*/*v*) isoflurane in spontaneously breathing mice [28]. Body temperature was monitored and maintained at 37 °C with a heating pad during all procedures. For analgesia, buprenorphine (Indivior, Slough, Berkshire, UK, 0.1 mg/kg body weight) was injected subcutaneously twice daily starting at the time of induction of SAH or sham surgery. SAH was induced under continuous monitoring of ICP, as described previously [28]. Animals were randomized to the SAH or sham group prior to surgery.

### Laser SPECKLE Contrast Imaging (LSCI)

**Fig. 1:**
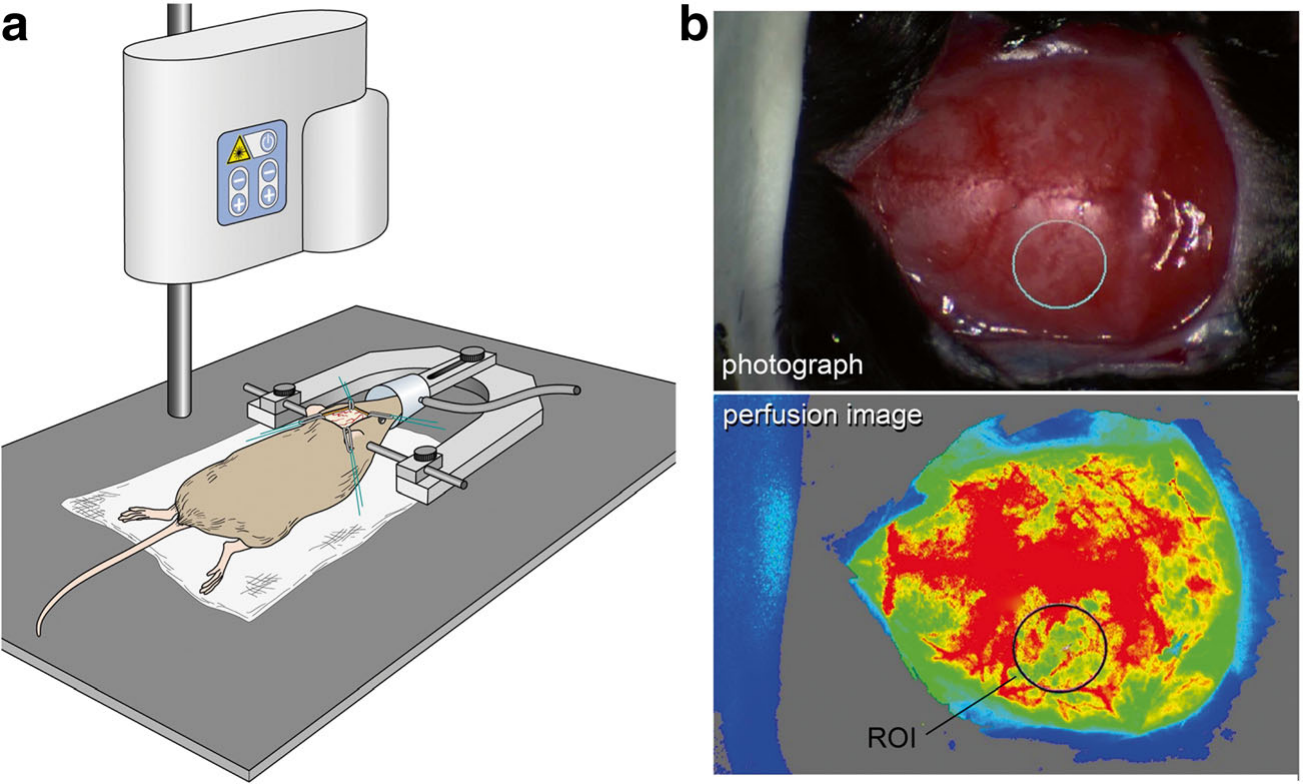
Determination of cerebral cortical perfusion. **a** The perfusion measurement: The animal is mounted on a stereotaxic frame. After the skin incision, the laser SPECKLE camera is placed over the animal to acquire perfusion images. **b** The evaluation of cerebral perfusion (upper image: photograph, lower image: flux image visualizing cerebral cortical perfusion). A region of interest of 7 mm^2^ is placed on the left parietal region to measure perfusion flux values

We used a laser perfusion imager (MoorFLPI-2-blood flow imager, Cologne, Germany) to visualize cerebral cortical perfusion of the whole convexity through the intact calvaria before induction of SAH and after 15 min, 3, 24, and 72 h. The animal’s skull was immobilized in a stereotaxic frame (Stoelting CO., IL, USA). A midline incision was made to expose the calvaria. Perfusion images and corresponding photographs were acquired every second for 60 s. After these measurements, the skin was closed using prolene 6.0 (Ethicon, Norderstedt, Germany). Figure 1 illustrates the experimental setting.

### Analysis of Cerebral Perfusion of the Left MCA Territory

LSCI data were evaluated using Moor review software (moorFLPI Full-Field Laser Perfusion Imager Review Version 4.0). A mean image was calculated from the 60 perfusion images. Mean flux values were determined from a region of interest (ROI) of 7 mm^2^ placed on the vascular territory of the left MCA [29] (see Fig. 1). Perfusion was evaluated by an investigator blinded to the treatment.

### Perfusion, Intravascular Casting, Microcomputed Tomography (Micro-CT) of Murine Brains, and Volumetric Analysis of CV

**Fig. 2:**
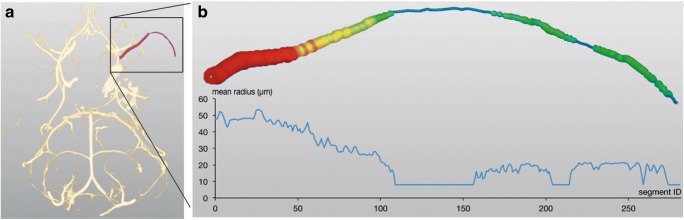
Determination of CV. **a**, **b** Determination of cerebral vessel volumes: the cerebrovascular tree is virtually reconstructed from Dicom data after micro-CT scanning of the brain (**a**). Afterwards, a defined vessel segment is selected and the vessel volume is calculated as a marker for CV (**b**)

After LSCI measurement 72 h after SAH, animals were sacrificed by a perfusion casting procedure with a radiopaque compound (Microfil MV-122, Flow Tech Inc., Carver, Massachusetts, USA), as previously described [28, 30]. Brain samples were scanned with a micro-CT (μCT40, Scanco Medical AG, Brüttisellen, Switzerland) with power settings of 70 kVp and 114 μA and a voxel size of 20 μm. Resulting DICOM data were imported to Amira® software, version 5.4.2 (FEI Visualization Sciences Group, Hillsboro, OR, USA). The intracranial vascular tree was reconstructed and the vessel volume of a defined 2.5-mm vessel segment of the MCA distal of the carotid T was calculated as previously described [28]. Volumetric analysis was selected on the basis of a methodological study, which compared vessel volumes and diameters in mice with SAH-induced vasospasm [28] and showed that the analysis of vessel diameters can determine vasospasm-induced vascular changes with a greater sensitivity. Figure 2 illustrates the method. CV was evaluated by an investigator blinded to the treatment.

### Statistics

Data are presented as mean ± standard error of the mean. The Mann–Whitney *U* test was used for statistical testing. Correlation analysis was calculated using Pearson’s correlation coefficients. The level of *p* < 0.05 was considered statistically significant. Group sizes were chosen after power analysis of pilot data (not shown) using Sigma Plot version 12.5 (Systat Software Inc., San Jose, CA, USA) with alpha of 0.05, power of 0.8, and expected differences standard deviations in vessel volumes of 0.003 ± 0.002 μL and in perfusion values of 25 ± 15% between SAH and sham groups.

## Results

### Murine Model of SAH

Fifteen animals were included. Five were assigned to the sham group and ten to the SAH group. Two SAH animals died (days 1 and 3 after SAH) and were thus excluded from analysis. Mean duration of surgery was similar between SAH and sham groups (SAH, 35.8 ± 3.0 min; sham, 37.0 ± 3.2 min). Baseline ICP was not significantly different between SAH and sham animals (SAH, 9.3 ± 1.8 mmHg; sham, 12.0 ± 2.6 mmHg). SAH induction resulted in a sharp increase in ICP with a peak at 71.8 ± 3.5 mmHg, whereas, during vascular insertion of the filament during sham surgery, we observed an ICP of 13.2 ± 2.1 mmHg. Loss of body weight was not significantly different between SAH and sham animals (before surgery, SAH 25.1 ± 0.5 g, sham, 26.4 ± 1.1 g; postop day 1, SAH 22.9 ± 0.7 g, sham, 23.8 ± 1.0 g; postop day 2, SAH 21.4 ± 0.7 g, sham, 22.8 ± 1.2 g; postop day 3, SAH 21.3 ± 0.6 g, sham, 22.7 ± 1.4 g). Neuroscores were higher for SAH animals (SAH vs. sham, 0.4 ± 0.4 vs. 0.0 ± 0.0 (d0); 5.6 ± 0.9 vs. 2 ± 0.4 (d1); 9.3 ± 1.6 vs. 5.8 ± 1.5 (d3)), although the difference reached statistical significance (*p* < 0.05) only on day 1. All of the SAH animals, but none of the sham animals, showed a subarachnoid hematoma.

### SAH Causes Cerebral Hypoperfusion

**Fig. 3:**
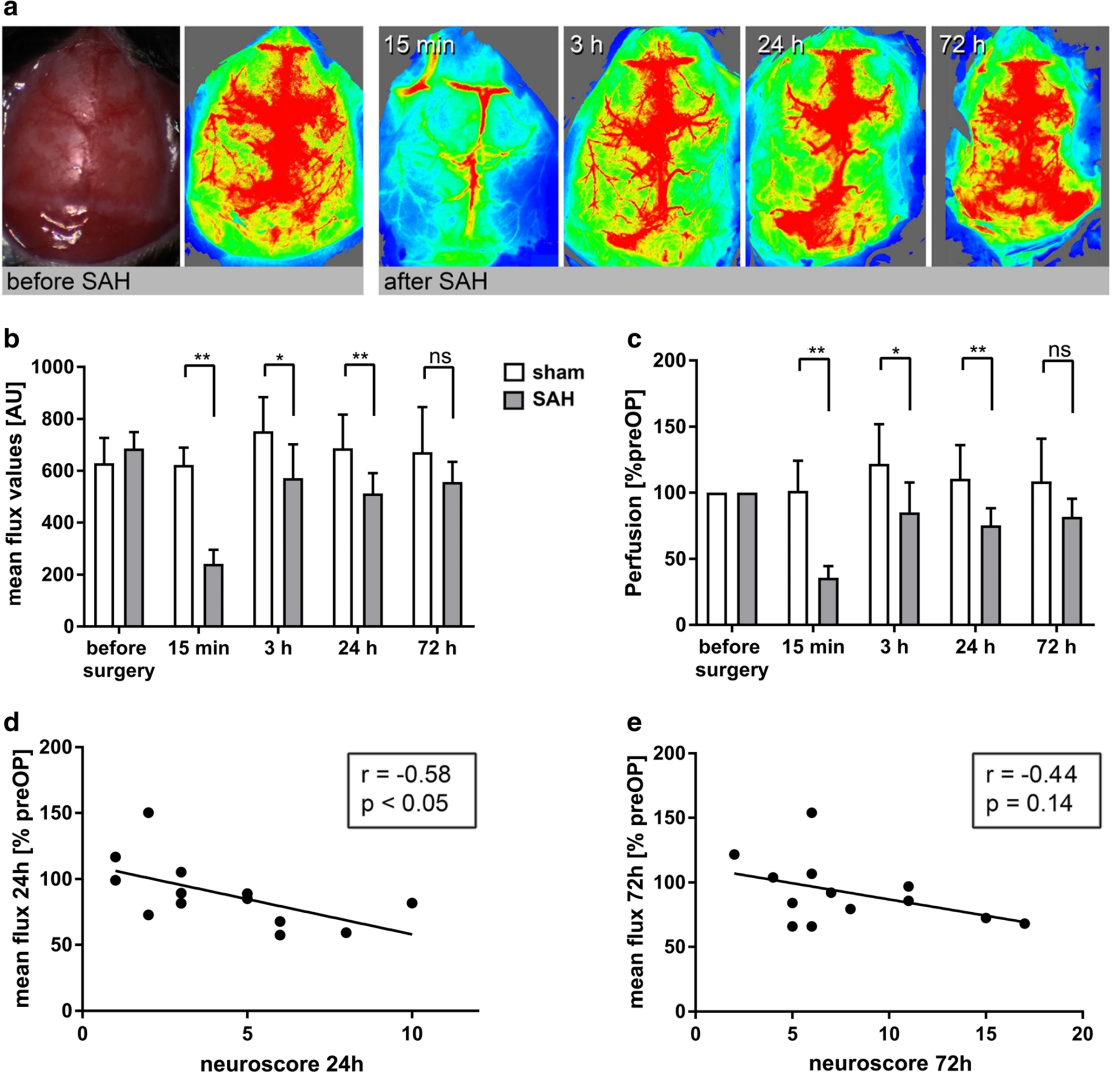
SAH induces cerebral hypoperfusion. **a** An anatomical image and representative perfusion images of the same animal before SAH and 15 min, 3, 24, and 72 h after SAH. **b**, **c** The course of cerebral perfusion after SAH and sham surgery. Note that perfusion is significantly impaired after SAH compared to sham (*p < 0.05, **p < 0.01). **d**, **e** The correlation between cerebral perfusion and neuroscore at 24 and 72 h

Flux values determined before induction of SAH were similar between SAH and sham animals. Induction of SAH induced a significant decrease in cortical perfusion in SAH animals compared to values before SAH (SAH, 35.7 ± 3.1%, sham, 101.4 ± 10.2%, *p* < 0.01). In SAH animals, perfusion partly recovered after 3, 24, and 72 h but remained significantly lower compared to that in sham (SAH vs. sham, 3 h, 85.0 ± 8.6 vs. 121.9 ± 13.4, *p* < 0.05; 24 h, 75.3 ± 4.6 vs. 110.6 ± 11.4%, *p* < 0.01; 72 h, 81.8 ± 4.8 vs. 108.5 ± 14.5%, n.s.; Fig. 3).

### SAH Induces Vasospasm

**Fig. 4:**
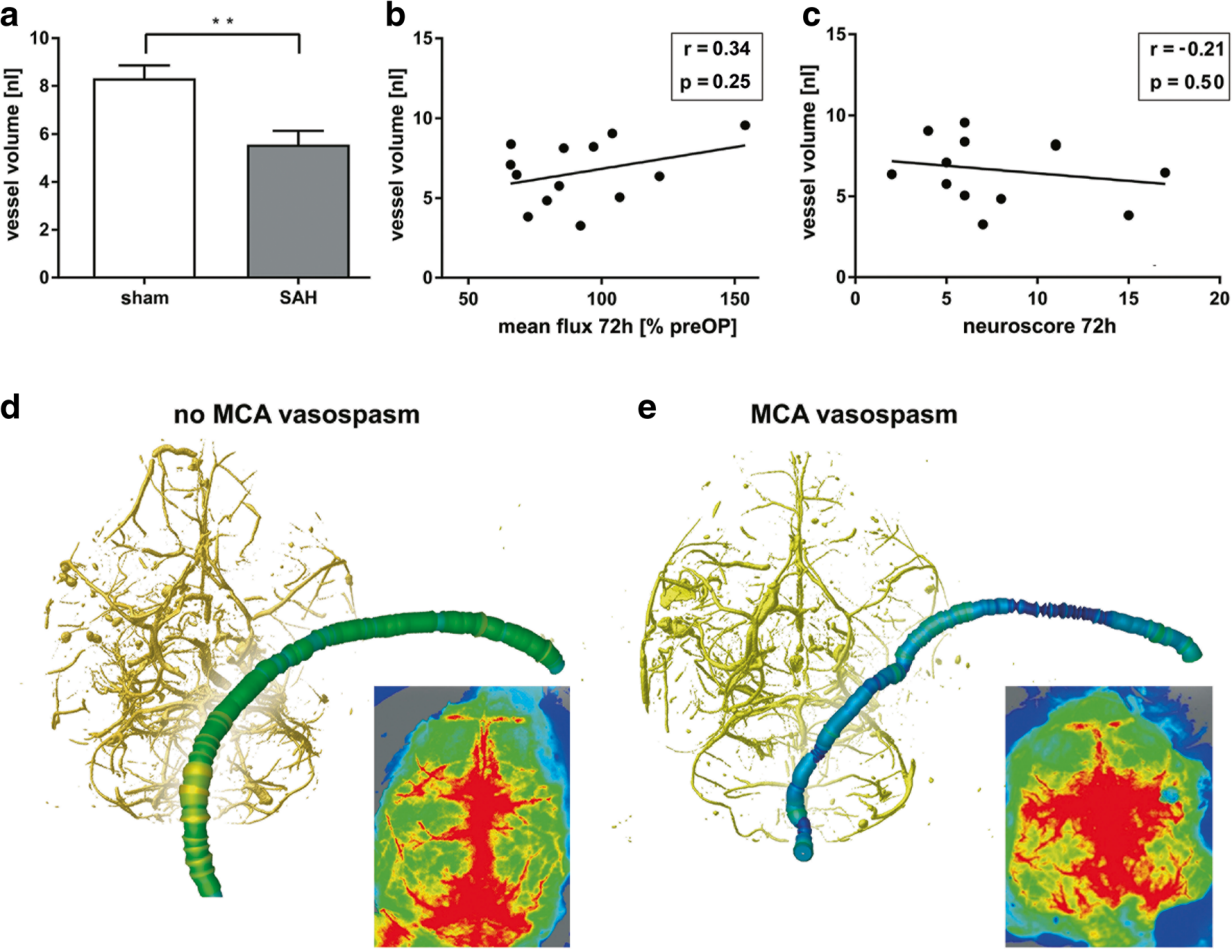
CV and cerebral perfusion after SAH. **a** Vessel volumes of a 2.5-mm segment of the MCA distal of the carotid T after SAH and sham surgery. Note that the vessel volumes are significantly lower in SAH animals, indicating CV. **b**, **c** The correlation of MCA vessel volume with perfusion and neuroscore. **d**, **e** Exemplarily show the reconstructed vascular tree and cerebral perfusion in a sample without CV (**d**) and with CV (**e**), in which CV was not associated with impaired perfusion

We assessed CV after perfusion and casting of the mice 72 h after SAH or sham surgery. This time point was selected to determine the impact on cortical perfusion at the peak of CV [16, 23, 24]. We analyzed the vessel volume of a 2.5-mm vessel segment of the left MCA distal of the carotid T, which is a highly sensitive parameter for evaluating vasospasm [28]. Vessel volumes of the left MCA were significantly lower in SAH animals compared to sham animals (SAH, 5.6 ± 0.6 nL; sham, 8.3 ± 0.5 nL, *p* < 0.01), indicating CV in the SAH group (Fig. 4).

### Correlation of Cerebral Perfusion, Vasospasm, and Neuroscore

Cerebral perfusion was determined in a ROI representing the vascular territory of the left MCA [29, 31]. To assess the influence of vasospasm on cerebral perfusion, we correlated vessel volumes of sham operated animals (normal vessel diameter) and SAH animals (vasospasm) with cortical perfusion images on day three. There was no significant correlation between the parameters (*r* = 0.34, *p* = 0.25). Interestingly, in some cases, pronounced MCA vasospasm was not associated with cortical hypoperfusion of the MCA territory (Fig. 4). To assess whether impaired cerebral perfusion and vasospasm were linked to neurological disability, we correlated cerebral perfusion with the corresponding neuroscore. There was a moderate correlation of perfusion with neuroscore (24 h: *r* = − 0.58, *p* < 0.05; 72 h: *r* = − 0.44, *p* = 0.14), which, however, did not reach statistical significance at 72 h (Fig. 3). There was no significant correlation between vessel volume and neuroscore (*r* = − 0.21, *p* = 0.50, Fig. 4).

In the next step, we used SAH data alone to determine if vessel diameter was correlated with cortical perfusion in animals with vasospasm to determine if differences in degree of vasospasm resulted in alterations of cortical perfusion. In this data set, no correlation between vasospasm, perfusion, and neuroscore was present. However, the power to assess this relationship may have been limited by the small group size and relatively high homogeneity in vessel volume between SAH animals.

## Discussion

To the best of our knowledge, this study was the first to analyze the relationship between CV, cerebral perfusion, and neurological impairment in a murine model of SAH. We determined cortical cerebral perfusion of the whole convexity through the intact calvaria using laser SPECKLE contrast imaging. In this way, we were able to analyze the course of cerebral perfusion at several time points within the first 72 h of SAH induction and to correlate perfusion of the MCA vascular territory with CV of the MCA and quantitative neuroscore. The most important findings of our study are (i) the lack of a clear correlation between CV and perfusion or neuroscore and (ii) the correlation between cortical perfusion and neuroscore, which became weaker at later time points. Taken together, these data indicate that factors other than CV play a major role in the alteration of cerebral perfusion after SAH, and that local cerebral malperfusion, rather than CV, determines neurological impairment.

In human patients, CV typically occurs within 14 days of SAH. As CV can lead to cerebral infarction, it is thought to be associated with a poor outcome [1–3]. Therefore, CV has become a key target of novel pharmaceutical agents in recent years. However, a number of studies achieved reduction of angiographic vasospasm without the expected improvement of clinical outcome [7–11]. In contrast, a study with nimodipine [32] achieved significant improvement in outcomes without significant changes in rate of CV. The interpretation of these findings is complicated because, in a clinical setting, factors such as adverse effects of pharmaceutical agents or rescue therapies could conceal the beneficial effect of amelioration of CV. Nevertheless, these clinical data imply that factors other than CV play an important role in outcome. Taking our experimental observations into account, neurological impairment appears to be linked to local changes in perfusion rather than to large vessel vasospasm of, for example, the MCA. Another important aspect of our study is that neurological impairment more weakly correlated with cortical perfusion at 72 h compared with that at 24 h. This could indicate that with increasing time after SAH, the role of cortical perfusion as a key driver of neurological disability declines. This is in line with clinical studies [4, 5], which have shown the high relevance of cerebral hypoperfusion early after SAH for unfavorable outcome. Therefore, future SAH treatment research should focus on causes of and strategies for cerebral perfusion, starting at an early time point after the bleeding event.

CV depicted on the angiogram is one factor, but not the primary factor, contributing to cerebral hypoperfusion. Studies conducted in recent years have identified additional factors that can occur independently of CV and impair cerebral microcirculation. In an autopsy series, Stein et al. observed microthrombi in patients after SAH [33]. In another study, Uhl et al. [34] described narrowing of microvessels early after SAH. These pathological changes at the level of microvessels were also recently described in a murine SAH model [21, 22]. Given that cerebrovascular resistance is primarily determined by vasoconstriction of microvessels [35], narrowing and thrombosis of microvessels has the potential to markedly affect cerebrovascular resistance and perfusion independently of CV. Therefore, we assume that increased cerebrovascular resistance caused by changes at the level of the microcirculation is the most likely factor responsible for cerebral hypoperfusion.

Finally, we want to address the limitations of this study. Cerebral perfusion is dependent on cerebral perfusion pressure. In line with data by Feiler et al. [27], we determined the two key factors of intracranial pressure and arterial blood pressure in a separate set of animals and did not find significant differences at days one and three after SAH. To limit the distress imposed upon the animals, we did not perform these analyses in the current study and so cannot present ICP and blood pressure data. We therefore cannot rule out the possibility that low cerebral perfusion pressure contributed to impaired cortical perfusion. Secondly, we measured cortical perfusion at different time points, whereas vasospasm was only determined 72 h after SAH. In addition, perfusion was examined in vivo, whereas vasospasm was analyzed ex vivo. The time point of 72 h was chosen because our data and other studies have reported that vasospasm peaks 72 h after induction of SAH in the murine endovascular filament perforation model [16, 23, 24]. Although we cannot rule out that a correlation between vasospasm and cortical perfusion may have been present at earlier time points, the lack of correlation at 72 h indicates that large vessel vasospasm is of limited relevance in the murine model. Third, it should be noted that perfusion levels exceeded the preoperative value after 3, 24, and 72 h in the sham group. These changes are most likely related to the isoflurane anesthesia, which is reported to have a cerebral vasodilatory effect [36]. It is therefore important to mention that the experimental conditions were similar between SAH and sham groups in this study. Finally, we only examined whether cortical perfusion correlated with vessel volume using sham animals for normal vessel diameter and SAH for vasospasm. Our study does not allow conclusions on whether different degrees of SAH-induced vasospasm correlate with hypoperfusion or neurological impairment. Although there are differences between the SAH animals, the model used here is designed to produce hemorrhage with high reproducibility. A larger number of animals would be required to assess correlations within the SAH group.

One last note of caution is warranted. Our data imply that CV is only of minor importance with regard to cerebral perfusion and neurological impairment in the murine model, suggesting that other factors play a prominent role, like elevated cerebrovascular resistance or microthrombi formation at the level of microcirculation. However, in contrast to humans, territorial infarctions as a result of CV after SAH occur only rarely in mice. This could indicate that, in the murine model, the contribution of CV to cerebral perfusion is less important, while the contribution of microcirculatory changes is more important, compared to human cases. Furthermore, in a clinical setting, aggressive treatment of CV may be the only option to improve cerebral hypoperfusion in cases of threatening cerebral infarction [1–3]. Therefore, although our findings underline the importance of future research efforts towards amelioration of cerebral perfusion by targeting CV-independent mechanisms, CV monitoring and, in some cases, aggressive treatment of CV remain a cornerstone of SAH therapy.
